# Supplementary material for: Clinical Significance of Tumor Size, Pathological Invasion Sites Including Urinary Collecting System and Clinically Detected Renal Vein Thrombus as Predictors for Recurrence in pT3a Localized Renal Cell Carcinoma
Source: Diagnostics (Basel). 2020 Mar 12;10(3):154. doi: 10.3390/diagnostics10030154 (PMC7151108; doi:10.3390/diagnostics10030154)
Supplement: Supplementary file 1 [file diagnostics-10-00154-s001.pdf]

Table S1. Correlation between size and Furhman grade/INF/growth pattern.

	**Spearman *r***	***p* Value**
**Furhman grade**	**0.91**	**<0.0001**
**INF**	**0.21**	**0.04**
**growth pattern**	**0.25**	**0.02**
